# Poliomyelitis

**Published:** 1911-02

**Authors:** W. H. Frost

**Affiliations:** Passed Assistant Surgeon, U. S. Public Health and Marine Hospital Service. Washington, D. C.


### CORRESPONDENCE

### Poliomyelitis.

**Is it of germ origin? Dr. Frost replies to our Editorial in the January Issue**

*Editor Buffalo Medical Journal:*

Sir—My attention has just been called to an editorial in the current (January, 1911), issue of your journal relative to a paper on "The field investigation of epidemic poliomyelitis" which was read by me at the last conference of Sanitary Officers of the State of New York in Buffalo, and published in the *Public Health Reports* of November 18, 1910.

Referring to two statements made in my paper—namely, "The disease is certainly due to a specific microörganism which can readily be destroyed by the usual methods of disinfection," and "It has been demonstrated that the specific causative organism is of minute size," your editorial makes the following criticism: "This language is positive and precise; it is either true or false; and it may be said of the statements that they are of enormous importance, if true. However, we are not aware of the announcement of even the first step of the demonstration of the germ origin of the neurotoxin of the so-called poliomyelitis."

I agree with you fully as to the importance of the facts referred to and the precision of the statements. The demonstrations upon which these statements are based are mentioned upon the same page of my paper, and are set forth in more detail in the first article cited among my references (Lovett, R. W., *Boston Medical and Surgical Journal,* Vol. 163, No. 2, p. 37,) where you may also find a bibliography of the most recent important work on poliomyelitis. However, since they have, by your own statement, escaped your notice, I beg leave to invite your attention to the announcements of some of the demonstrations which have established the germ origin of epidemic poliomyelitis.

The first satisfactory proof that epidemic poliomyelitis is due to an infectious organism was presented by Flexner and Lewis of the Rockefeller Institute for Medical Research, in the *Journal of the American Medical Association,* November 13, 1909, p. 1639. The authors report there the experimental production of poliomyelitis in a monkey by inoculation of an emulsion of the cord of a child who had died of the disease. Portions of the cord and brain of this monkey inoculated into other monkeys caused the disease, which was then similarly transferred from these to still other monkeys. From these experiments the authors conclude (referring to epidemic poliomyelitis) that "any doubt of its infectious origin can hardly be longer maintained."

In a later issue of the *Journal of the American Medical Association* (December 18, 1909, p. 2095) Flexner and Lewis reported that they had been able to produce the disease in monkeys by inoculation of an emulsion of virulent spinal cord filtered through a filter (Berkefeld) of sufficient fineness to prevent the passage of bacteria. Similar results were previously and independently obtained by Landsteiner and Levaditi in the Pasteur Institute of Paris and later by several others (*Compt. rend. de la Soc. de Biol.,* November 27, 1909, and December 18, 1909).

The statement that the virus is readily destroyed by the usual methods of disinfection is based upon the following experiments:

Flexner and Lewis found that a temperature of 40 degrees to 50 degrees C., maintained for half an hour, rendered the virus inactive. They also found (*Journ. Am. Med. Assn.,* May 28, 1910, p. 1782) that a dilution of perhydrol containing 1 per cent. of hydrogen peroxide rendered the virus inactive.

Landsteiner and Levaditi (*Compt. rend. de la Soc. Biol.,* March 5 and May 30, 1910) have shown that the virus is rendered inactive by the following disinfectants: perhydrol, Merck, 1 : 5 dilution, mixed with an equal volume of an emulsion of spinal cord, and allowed to stand 45 minutes at 37 degrees C.; permanganate of potash, 1 : 500 mixed with an equal volume of emulsion and allowed to stand 1 hour; a powder of menthol 0.2, salol 5, and boric acid 20, 0.05 cc. of the above powder being mixed with 2 cc. of an emulsion of the virus and allowed to stand for 2 hours.

Römer and Josepa (*Munchener Med. Woch.,* May 17, 1910, vol. 57, No. 20, p. 946) have shown that the virus may be destroyed by "the usual process of formaldehyde disinfection."

A review of the many experiments demonstrating the infectious origin of epidemic poliomyelitis would be altogether too voluminous to undertake in a letter and is fortunately quite unnecessary, since I need only refer you to an article by Dr. Simon Flexner in the *Journal of the American Medical Association* of September 24, 1910, vol. 55, pp. 1105-1113, where you may find the subject clearly and concisely reviewed. From Dr. Flexner's article I quote the following:

> These methods led to the discovery almost simultaneously in the United States by Dr. Lewis and myself and in France by Landsteiner and Levaditi that the infectious agent is *an extremely minute microörganism* that readily passes through the pores of earthenware filters and constitutes, therefore, an example of the so-called filterable viruses, of which at the present time several varieties are known to cause infectious diseases in man and the lower animals.

"The virus causing epidemic poliomyelitis has been stated to be a very minute size. It is, so far as we can now judge, one of the most minute organisms known to cause disease. This conclusion follows from the fact that in aqueous suspension . . . . . it passes with great readiness and little or no loss of potency through the pores of the densest and finest porcelain filters,—namely, the so-called Chamberlain filter. . . . . .The filtrates are highly potent. Quantities as small as one one-thousandth to one one-hundredth of a cubic centimeter suffice to cause paralysis in monkeys after the usual incubation period when injected into the brain.

That the *virus is a living organism* must be concluded from the fact that such minute quantities of it suffice to carry infection through an indefinite series of animals. We have propagated the virus now through twenty-five generations, representing twenty-five separate series of monkeys.

I enclose for your further information a list of references to some of the more important articles dealing with the experimental transmission of epidemic poliomyelitis, trusting that all the articles may be available to you, since they constitute a most interesting chapter in the history of the progress of medical science.

Your editorial credits me with making the "statement that 'virus' and 'germ' are synonymous terms" and with the "dogmatic assumption that where there is a virus there must be a germ, or where there is a germ there must be a virus." The only sentence in which I have used the two words "virus" and "germ" reads as follows: "In the bodies of infected animals the virus (germ) of the disease has been demonstrated not only in the spinal cord and brain, but in the nasal mucous membrane," etc. This sentence conveys (as was my intention) the obvious inference that the virus of poliomyelitis is a germ; it does not enter into any discussion of the general synonym and coextensiveness of the two terms,—a matter which may be left to the makers of dictionaries. You may note, in this connection, that Dr. Flexner in the above cited quotation has definitely asserted that the virus of poliomyelitis is a living organism, which I take to be equivalent to stating that it is a "germ" in the generally accepted sense of the word.

I should greatly appreciate the courtesy if you will publish this letter in the next issue of your journal. The demonstration of the infectious nature of epidemic poliomyelitis has obviously an important bearing upon its prevention, and I should consider it a privilege to be allowed to place before your readers the facts upon which is based the positive and precise assertion that the disease is due to a specific microörganism.

Thanking you for your commendation of the motive of my article,

Yours very truly,

W. H. FROST,

*Passed Assistant Surgeon,*

*U. S. Public Health and Marine Hospital Service.*

Washington, D. C., January 5, 1911.

EXPERIMENTAL POLIOMYELITIS.

(REFERENCES, 1909-1910. PARTIAL LIST.)

Flexner, Simon, Journ. Am. Med. Assn., 1910, Vol. 55, p. 1105-1113; Pediatrics, 1910, Vol. 22, No. 8, p. 477-486; Johns Hopkins Hosp. Bull., 1910, vol. 21, p. 118.

Flexner, Simon and Lewis, Paul A., Journ. Am. Med. Assn., 1909, Vol. 53, p. 1639; p. 1953; p. 2093; Journ. Am. Med. Assn., 1910, Vol. 54, p. 45; 535; 1140; 1780; Journ. Am. Med. Assn., 1910, Vol. 55, p. 662-663; Journ. Exper. Med., 1910, Vol. 12, p. 227-235.

Kraus, Rudolph, Med. Klin., 1910, Vol. 6, p. 470-472.

Krause, P. and Meinicke, E., Deut. Med. Woch., 1910, Vol. 35, p. 647; p. 1825.

Levaditi, C., and Stanesco, V., Compt. Rend. de la Soc. de Biol., 1910, Vol. 68, p. 311.

Levaditi, C., and Landsteiner, K., Ann. de l'Inst. Pasteur, 1910, Vol. 24, p. 833-876.

Leiner, C., and Weisner, R. V., Wien. Klin. Woch., 1909, Vol. 22, p. 1698-1701; Wien. Klin. Woch., 1910, Vol. 23, p. 91; 323; 817.

Romer, P., and Joseph H., Munch, Med. Woch., 1910, Vol. 57, p. 347; 520; 568; 945; 1059.

EDITOR'S NOTE.—We print the foregoing with pleasure and would simply call the attention of our readers to the fact that Dr. Frost is becoming more and more involved. His citations, which are not new to us, merely indicate that observers hold that the disease in question is infectious, a matter not at issue; again, that other observers have faith, opinions, believe that they are warranted in the inference that the disease is of germ origin.

Our impression remains as before, that scientific demonstrations are beyond the realms of probable opinions, or of reasonable inferences. Dr. Frost's citations would seem to assert, if they assert anything, that there is no infection without a causative germ, that there is no virus without the same germ. We still believe that the victim of the bite of the cobra, or the spider, or the sting of the bee, has a distinct infection without germ causation; and also, that we have stated the matter with a due regard to the findings of recent investigations when we assert our belief that the so-called poliomyelitis is the result of a virulent neurotoxin,—an enzymic poison.

The most unfortunate mental attitude of our esteemed correspondent is his virtual assertion that Flexner demonstrated the germ origin of so-called poliomyelitis, when he announced his hope that some day the germ origin of scarlet fever, yellow fever, smallpox, and rabies might be demonstrated. We shall be only too willing to proclaim these important discoveries upon their announcement, and in the meantime will try and remember that hopes and scientific demonstrations frequently arrive at widely separated periods of study.

